# IUC24424-81 Real-world efficacy and safety of maintenance avelumab in locally advanced or metastatic urothelial cancer

**DOI:** 10.1093/oncolo/oyaf248.027

**Published:** 2025-08-29

**Authors:** O Okuma, O Olatunji, E C Okuofo

**Affiliations:** The James Cook University Hospital, United Kingdom; The James Cook University Hospital, United Kingdom; The James Cook University Hospital, United Kingdom

## Abstract

**Background:**

Avelumab is approved for use as first-line maintenance therapy (1LM) in locally advanced and metastatic urothelial cancer, after first-line platinum-based chemotherapy (1LPBC), following its demonstrable benefits in the JAVELIN Bladder 100 phase 3 trial. However, the outcomes in the real-world setting are yet to be extensively evaluated. This study evaluates the real-world efficacy and safety of 1LM Avelumab.

**Methods:**

This is a single-center retrospective study of all patients with locally advanced or metastatic urothelial cancer treated with 1LM avelumab after no progression following 1LPBC. The period of study was from January 2022 to December 2024. A total of 38 patients were involved in this study. Data were collected and analyzed using Excel and SPSS version 26. A *P*-value of .05 was selected for statistical significance.

The Primary Objective was to evaluate OS at 12 months. Secondary objectives were median overall survival (OS), median PFS, and incidence of ≥ Grade 3 toxicities.

**Results:**

The median age was 71.5 years (95% CI: 70-76), and the population consisted of 71.1% males and 28.9% females. About 97.4% had a performance status of 0-1, 60.5% had complete or partial response following 1LPBC (vs 72.3% in the JAVELIN trial), while 39.5% had stable disease (vs 27.7% in JAVELIN). The OS at 12 months was 52.6% with a median value of 14 months (95% CI, 8.6-19.7), compared to 71.3% and a median value of 21.4 months (95% CI, 18.9-26.1) in the JAVELIN trial. Linear regression analysis showed a statistically significant relationship between age and OS (*P*-value = .006). Median PFS was 7 months (95% CI, 3-11), compared to 3.7 months (95% CI, 3.5-5.5) in the JAVELIN trial. Only 13.2% experienced >/= Grade 3 toxicities.

**Conclusions:**

Our study showed a better median PFS compared to the original Javelin study. However, the OS trumped in comparison. This may be related to an older cohort of patients and a higher proportion of patients with stable disease in response to first-line platinum-based chemotherapy in our study, which is reflective of real-world experience.

